# A new family of *q*-hypergeometric congruences from Andrews’ multi-series transformation

**DOI:** 10.1007/s13398-025-01721-4

**Published:** 2025-04-10

**Authors:** Victor J. W. Guo, Michael J. Schlosser

**Affiliations:** 1 School of Mathematics, Hangzhou Normal University, Hangzhou, 311121 People’s Republic of China; 2 Fakultät für Mathematik, Universität Wien, Oskar-Morgenstern-Platz 1, 1090 Vienna, Austria

**Keywords:** Basic hypergeometric series, Supercongruences, *q*-Congruences, Cyclotomic polynomial, Andrews’ transformation, Gasper’s summation, Primary 33D15, Secondary 11A07, 11B65

## Abstract

We deduce a new family of *q*-hypergeometric congruences modulo the fourth power of a cyclotomic polynomial from George Andrews’ multi-series extension of the Watson transformation. A Karlsson–Minton type summation for very-well-poised basic hypergeometric series due to George Gasper also plays an important role in our proof. We put forward two relevant conjectures on supercongruences and *q*-supercongruences for further study.

## Introduction

Let $(a)_n=a(a+1)\cdots (a+n-1)$ denote the rising factorial, or the Pochhammer symbol. In 2016, Long and Ramakrishna [19, Thm. 2] established the following supercongruence: for any odd prime *p*,

1.1 $$\begin{aligned} \sum _{k=0}^{p-1} (6k+1) \frac{(\frac{1}{3})_k^6}{k!^6} \equiv {\left\{ \begin{array}{ll} -p \Gamma _p(\tfrac{1}{3})^9 \pmod {p^6}, & \text {if}\ p\equiv 1\pmod 6,\\ -\frac{10p^4}{27} \Gamma _p(\tfrac{1}{3})^9\pmod {p^6}, & \text {if}\ p\equiv 5\pmod 6, \end{array}\right. } \end{aligned}$$

where $\Gamma _p(x)$ denotes the *p*-adic Gamma function. For $p\equiv 1\pmod 6$ this result confirms Van Hamme’s (D.2) supercongruence [24], which is a congruence modulo $p^4$. The supercongruence (1.1) is now called a Ramanujan-type supercongruence. For more such supercongruences, we refer the reader to [24, 27].

Nowadays, a number of supercongruences have been generalized to *q*-supercongruences by different authors (see, for example, [7, 9, 11, 12, 16, 25]). In particular, the authors [11, Theorem 2.3] gave a partial *q*-analogue of (1.1):

1.2 $$\begin{aligned} \sum _{k=0}^{n-1} [6k+1]\frac{(q;q^3)_k^6}{(q^3;q^3)_k^6} q^{3k} \equiv {\left\{ \begin{array}{ll} 0 \pmod {[n]}, & \text {if}\ n\equiv 1\pmod 3,\\ 0 \pmod {[n]\Phi _n(q)}, & \text {if}\ n\equiv 2\pmod 3. \end{array}\right. } \end{aligned}$$

Shortly afterwards, they [13, 14] further proved the following result: for any integers *d*, *r*, *n* satisfying $d\geqslant 2$, $r\leqslant d-2$, $n\geqslant d-r$, *d* and *r* are coprime, and $n\equiv -r\pmod {d}$, there holds

1.3 $$\begin{aligned} \sum _{k=0}^{n-1}[2dk+r] \frac{(q^r;q^d)_k^{2d}}{(q^d;q^d)_k^{2d}}q^{d(d-1-r)k} \equiv 0 \pmod {[n]\Phi _{n}(q)^3}. \end{aligned}$$

Recently, Wei [25] obtained the following complete *q*-analogue of the second supercongruence in (1.1): for any positive integer $n\equiv 2\pmod 3$,

1.4 $$\begin{aligned} \sum _{k=0}^{n-1}[6k+1]\frac{(q;q^3)_k^6}{(q^3;q^3)_k^6}q^{3k}&\equiv 5[2n] \frac{(q^2;q^3)_{(2n-1)/3}^3}{(q^3;q^3)_{(2n-1)/3}^3} \pmod {[n]\Phi _n(q)^5}, \end{aligned}$$

He also generalized (1.2) for $n\equiv 1\pmod 3$ to the modulus $[n]\Phi _n(q)^4$ case. At this point, it is appropriate to recall the standard *q*-notation (see [3]). For any indeterminates *a* and *q*, let $(a;q)_n=(1-a)(1-aq)\cdots (1-aq^{n-1})$ be the *q*-*shifted factorial*. For brevity, we adopt the notation $(a_1,a_2,\ldots ,a_m;q)_n=(a_1;q)_n (a_2;q)_n\cdots (a_m;q)_n$. Moreover, $[n]=[n]_q=1+q+\cdots +q^{n-1}$ denotes the *q*-*integer* and $\Phi _n(q)$ the *n*-th *cyclotomic polynomial* in *q*, which is irreducible over the integers and may be factorized over the complex numbers as

$$\begin{aligned} \Phi _n(q)=\prod _{\begin{array}{c} 1\leqslant k\leqslant n\\ \gcd (n,k)=1 \end{array}}(q-\zeta ^k), \end{aligned}$$

where $\zeta$ is any *n*-th primitive root of unity.

In this paper, we shall establish the following generalization of (1.3).

### Theorem 1.1

Let *d*, *r*, *s*, *n* be integers satisfying $d\geqslant 2$, $r\leqslant d-2$ (in particular, *r* is possibly negative), $0\leqslant s\leqslant d-r-2$, and $n\geqslant d-r$, such that $\gcd (d,r)=1$ and $n\equiv -r\pmod {d}$. Then

1.5 $$\begin{aligned} \sum _{k=0}^{M}[2dk+r]_{q^2} [2dk+r]^{2s} \frac{(q^{2r};q^{2d})_k^{2d}}{(q^{2d};q^{2d})_k^{2d}}q^{2d(d-r-s-1)k} \equiv 0 \pmod {[n]_{q^2}\Phi _{n}(q^2)^3},\nonumber \\ \end{aligned}$$

where $$M=(dn-n-r)/d$$ or $$M=n-1$$.

Letting $n=p^m$ be a prime power and then taking the limits as $q\rightarrow 1$, we get the following supercongruence from (1.5): for any integers *d*, *r*, *s* with $d\geqslant 2$, $r\leqslant d-2$, $0\leqslant s\leqslant d-r-2$ and $\gcd (d,r)=1$, and prime *p* with $p^m\geqslant d-r$ and $p^m\equiv -r\pmod {d}$,

1.6 $$\begin{aligned} \sum _{k=0}^{M}(2dk+r)^{2s+1} \frac{(\frac{r}{d})_k^{2d}}{(1)_k^{2d}} \equiv 0 \pmod {p^{m+3}}, \end{aligned}$$

where $$M=(dp^m-p^m-r)/d$$ or $$M=p^m-1$$.

The proof of Theorem 1.1 is similar to that of (1.3). Namely, we need to make a careful use of Andrews’ multi-series generalization (2.2) of Watson’s $_8\Phi _7$ transformation [1, Theorem 4], along with Gasper’s very-well-poised Karlsson–Minton type summation [2, Eq. (5.13)]. We remark that Andrews’ transformation plays an important part in number theory and combinatorics. For instance, Zudilin [26] utilized Andrews’ transformation to solve a problem of Schmidt. Krattenthaler and Rivoal [18] applied it to give an alternative proof of a result of Zudilin relating a very-well-poised hypergeometric series to a Vasilenko–Vasilev-type multiple integral, an important tool in the study of the arithmetic behavior of values of the Riemann zeta function at integers. The couple Hessami Pilehrood [17] also employed this transformation to provide a short proof of a theorem of   Zagier.   For the application of Andrews’ multi-series transformation in *q*-congruences, see [4–9, 12, 15, 23].

We shall prove the *q*-congruence (1.5) modulo $\Phi _n(q^2)^4$ in Sect. 2, and prove it is also true modulo $[n]_{q^2}$ in Sect. 3. The combination of these two congruences results in a congruence modulo the least common multiple of $\Phi _n(q^2)^4$ and $[n]_{q^2}$, i.e., modulo $[n]_{q^2}\Phi _n(q^2)^3$, which full establishes Theorem 1.1. Finally, we propose two related conjectures in Sect. 4.

## Proof of (1.5) modulo $\Phi _n(q^2)^4$

We need a simple *q*-congruence modulo $\Phi _n(q^2)^2$, which is intrinsically the same as [13, Lemma 1]. For the reader’s convenience, we give a new proof here.

### Lemma 2.1

Let $\alpha$, *r* be integers and *n* a positive integer. Then

2.1 $$\begin{aligned} (q^{2r-2\alpha n},q^{2r+2\alpha n};q^{2d})_k \equiv (q^{2r};q^{2d})_k^2 \pmod {\Phi _n(q^2)^2}. \end{aligned}$$

### Proof

It is easy to see that

$$\begin{aligned} (aq^{2r},bq^{2r};q^{2d})_k-(q^{2r},abq^{2r};q^{2d})_k \equiv 0\pmod {(1-a)(1-b)}. \end{aligned}$$

Since $1-q^{\pm 2n}\equiv 0\pmod {\Phi _n(q^2)}$, putting $a=q^{-2\alpha n}$ and $b=q^{2\alpha n}$ in the above congruence, we immediately get the desired *q*-congruence (2.1). $\square$

We will make use of a wonderful transformation formula due to Andrews [1, Theorem 4], which can be written as follows:

2.2 $$\begin{aligned}&\sum _{k\geqslant 0}\frac{(a,q\sqrt{a},-q\sqrt{a},b_1,c_1,\dots ,b_m,c_m,q^{-N};q)_k}{(q,\sqrt{a},-\sqrt{a},aq/b_1,aq/c_1,\dots ,aq/b_m,aq/c_m,aq^{N+1};q)_k} \left( \frac{a^mq^{m+N}}{b_1c_1\cdots b_mc_m}\right) ^k \nonumber \\&\quad =\frac{(aq,aq/b_mc_m;q)_N}{(aq/b_m,aq/c_m;q)_N} \sum _{j_1,\dots ,j_{m-1}\geqslant 0} \frac{(aq/b_1c_1;q)_{j_1}\cdots (aq/b_{m-1}c_{m-1};q)_{j_{m-1}}}{(q;q)_{j_1}\cdots (q;q)_{j_{m-1}}} \nonumber \\&\qquad \times \frac{(b_2,c_2;q)_{j_1}\dots (b_m,c_m;q)_{j_1+\dots +j_{m-1}}}{(aq/b_1,aq/c_1;q)_{j_1} \dots (aq/b_{m-1},aq/c_{m-1};q)_{j_1+\dots +j_{m-1}}} \nonumber \\&\qquad \times \frac{(q^{-N};q)_{j_1+\dots +j_{m-1}}}{(b_mc_mq^{-N}/a;q)_{j_1+\dots +j_{m-1}}} \frac{(aq)^{j_{m-2}+\dots +(m-2)j_1} q^{j_1+\dots +j_{m-1}}}{(b_2c_2)^{j_1}\cdots (b_{m-1}c_{m-1})^{j_1+\dots +j_{m-2}}}. \end{aligned}$$

This transformation can be deemed a multi-series generalization of Watson’s $_8\phi _7$ transformation formula (see [3, Appendix (III.18)]) which we state below in standard notation for *q*-series [3, Section 1] (*n* being a non-negative integer):

2.3 $$\begin{aligned}&_{8}\phi _{7}\!\left[ \begin{array}{cccccccc} a,&  qa^{\frac{1}{2}},&  -qa^{\frac{1}{2}}, &  b, &  c, &  d, &  e, &  q^{-n} \\ &  a^{\frac{1}{2}}, &  -a^{\frac{1}{2}}, &  aq/b, &  aq/c, &  aq/d, &  aq/e, &  aq^{n+1} \end{array};q,\, \frac{a^2q^{n+2}}{bcde} \right] \nonumber \\&\quad =\frac{(aq, aq/de;q)_n}{(aq/d, aq/e;q)_n} \, _{4}\phi _{3}\!\left[ \begin{array}{c} aq/bc,\ d,\ e,\ q^{-n} \\ aq/b,\, aq/c,\, deq^{-n}/a \end{array};q,\, q \right] . \end{aligned}$$

Next, we require the following very-well-poised Karlsson–Minton type summation due to Gasper [2, Eq. (5.13)] (see also [3, Ex. 2.33 (i)]):

2.4 $$\begin{aligned}&\sum _{k=0}^\infty \frac{(a,q\sqrt{a},-q\sqrt{a},b,a/b,d,e_1,aq^{n_1+1}/e_1,\dots , e_m,aq^{n_m+1}/e_m;q)_k}{(q,\sqrt{a},-\sqrt{a},aq/b,bq,aq/d,aq/e_1,e_1q^{-n_1}, \dots ,aq/e_m,e_mq^{-n_m};q)_k}\left( \frac{q^{1-\nu }}{d}\right) ^k\nonumber \\&\quad =\frac{(q,aq,aq/bd,bq/d;q)_\infty }{(bq,aq/b,aq/d,q/d;q)_\infty } \prod _{j=1}^m\frac{(aq/be_j,bq/e_j;q)_{n_j}}{(aq/e_j,q/e_j;q)_{n_j}}, \end{aligned}$$

where $n_1,\dots ,n_m$ are non-negative integers, $\nu =n_1+\cdots +n_m$, and $|q^{1-\nu }/d|<1$ if the series does not terminate. An elliptic extension of the terminating $d=q^{-\nu }$ case of (2.4) was given by Rosengren and the second author [22, Eq. (1.7)].

It is worth mentioning that the right-hand side of (2.4) vanishes for $d=bq$. Additionally, putting $b=q^{-N}$ we get the following terminating summation:

2.5 $$\begin{aligned} \sum _{k=0}^N\frac{(a,q\sqrt{a},-q\sqrt{a},e_1,aq^{n_1+1}/e_1,\dots , e_m,aq^{n_m+1}/e_m,q^{-N};q)_k}{(q,\sqrt{a},-\sqrt{a},aq/e_1,e_1q^{-n_1}, \dots ,aq/e_m,e_mq^{-n_m},aq^{N+1};q)_k}q^{(N-\nu )k}=0,\nonumber \\ \end{aligned}$$

which is valid for $N>\nu =n_1+\cdots +n_m$.

By properly combining (2.2) with (2.5), we can establish the following multi-series summation formula, which is a generalization of [13, Lemma 2].

### Lemma 2.2

Let $m\geqslant 2$ and $0\leqslant s\leqslant m-1$. Let *q*, *a* and $e_{s+1},\dots ,e_{m+1}$ be arbitrary parameters with $e_{m+1}=e_{s+1}$, and let $n_1,\dots ,n_m$ and *N* be non-negative integers satisfying $N>n_1+\cdots +n_m$. Then

2.6 $$\begin{aligned}&0=\sum _{j_1,\dots ,j_{m-1}\geqslant 0}\nonumber \\&\frac{(q^{-n_1};q)_{j_1}\cdots (q^{-n_s};q)_{j_s}(e_{s+1}q^{-n_{s+1}}/e_{s+2};q)_{j_{s+1}}\cdots (e_{m-1}q^{-n_{m-1}}/e_m;q)_{j_{m-1}}}{(q;q)_{j_1}\cdots (q;q)_{j_{m-1}}} \nonumber \\&\quad \times \frac{(\sqrt{a}q^{\frac{n_2+1}{2}},\sqrt{a}q^{\frac{n_2+1}{2}};q)_{j_1}\cdots (\sqrt{a}q^{\frac{n_s+1}{2}},\sqrt{a}q^{\frac{n_s+1}{2}};q)_{j_1+\cdots +j_{s-1}}}{(\sqrt{a}q^{\frac{1-n_1}{2}},\sqrt{a}q^{\frac{1-n_1}{2}};q)_{j_1}\cdots (\sqrt{a}q^{\frac{1-n_{s-1}}{2}},\sqrt{a}q^{\frac{1-n_{s-1}}{2}};q)_{j_1+\cdots +j_{s-1}}} \nonumber \\&\quad \times \frac{(aq^{n_{s+1}+1}/e_{s+1},e_{s+2};q)_{j_1+\cdots +j_s}\dots (aq^{n_m+1}/e_m,e_{m+1};q)_{j_1+\dots +j_{m-1}}}{(e_sq^{-n_s},aq/e_{s+1};q)_{j_1+\cdots +j_s} \dots (e_{m-1}q^{-n_{m-1}},aq/e_m;q)_{j_1+\dots +j_{m-1}}} \nonumber \\&\quad \times \frac{(q^{-N};q)_{j_1+\dots +j_{m-1}}}{(e_1q^{n_m-N+1}/e_m;q)_{j_1+\dots +j_{m-1}}(aq^{n_2})^{j_1}\cdots (aq^{n_s})^{j_1+\cdots +j_{s-1}}} \nonumber \\&\quad \times \frac{(aq)^{j_{m-2}+\dots +(m-2)j_1} q^{j_1+\dots +j_{m-1}}}{(aq^{n_{s+1}+1}e_{s+2}/e_{s+1})^{j_1+\cdots +j_s}\cdots (aq^{n_{m-1}+1}e_m/e_{m-1})^{j_1+\dots +j_{m-2}}}. \end{aligned}$$

### Proof

Performing the parameter substitutions $b_i=c_i=\sqrt{a}q^{\frac{1+n_i}{2}}$ for $1\leqslant i\leqslant s$, and $b_i\mapsto aq^{n_i+1}/e_i$, $c_i\mapsto e_{i+1}$, for $s+1\leqslant i\leqslant m$ (where $e_{m+1}=e_{s+1}$) in the multisum transformation (2.2), and dividing both sides by the prefactor of the multisum, we see that the series on the right-hand side of (2.6) is equal to

$$\begin{aligned}&\frac{(e_mq^{-n_m},aq/e_{s+1};q)_N}{(aq,e_mq^{-n_m}/e_{s+1};q)_N}\\&\qquad \times \sum _{k=0}^N\frac{(a,q\sqrt{a},-q\sqrt{a},e_1,aq^{n_1+1}/e_1,\dots , e_m,aq^{n_m+1}/e_m,q^{-N};q)_k}{(q,\sqrt{a},-\sqrt{a},aq/e_1,e_1q^{-n_1}, \dots ,aq/e_m,e_mq^{-n_m},aq^{N+1};q)_k}q^{(N-\nu )k}, \end{aligned}$$

with $e_i=\sqrt{a}q^{\frac{1+n_i}{2}}$ for $1\leqslant i\leqslant s$ and $\nu =n_1+\cdots +n_m$. Now the last sum vanishes by the special case (2.5) of Gasper’s summation. $\square$

We have gathered enough ingredients and are able to prove the *q*-congruence (1.5) modulo $\Phi _n(q^2)^4$.

### Proof of (1.5)

modulo $\Phi _n(q^2)^4$ For $M=(dn-n-r)/d$, the left-hand side of (1.5) can be written as the following multiple of a terminating $_{2d+2s+4}\phi _{2d+2s+3}$ series:

$$\begin{aligned} [2r] \sum _{k=0}^{(dn-n-r)/d}&\left( \frac{(q^{2r},q^{2d+r},-q^{2d+r},q^{2d+r},\ldots ,q^{2d+r}, q^{2r},\ldots ,q^{2r};q^{2d})_k}{(q^{2d},q^{r},-q^{r},q^r,\ldots ,q^r,q^{2d},\ldots ,q^{2d};q^{2d})_k}\right. \\&\quad \left. \times \frac{(q^{2d+2(d-1)n},q^{2r-2(d-1)n};q^{2d})_k}{(q^{2r-2(d-1)n},q^{2d+2(d-1)n};q^{2d})_k} q^{2d(d-r-s-1)k} \right) . \end{aligned}$$

Here, the $q^{2d+r},\ldots ,q^{2d+r}$ in the numerator means 2*s* instances of $q^{2d+r}$, the $q^{2r},\ldots ,q^{2r}$ in the numerator refers to $2d-1$ instances of $q^{2r}$. Similarly, the $q^{r},\ldots ,q^{r}$ in the denominator means 2*s* instances of $q^{r}$, and the $q^{2d},\ldots ,q^{2d}$ in the denominator refers to $2d-1$ instances of $q^{2d}$.

Now, by the $m=d+s$ case of Andrews’ transformation (2.2), we can write the above expression as

2.7 $$\begin{aligned}&[2r]\frac{(q^{2(d+r)},q^{-2(d-1)n};q^{2d})_{(dn-n-r)/d}}{(q^{2d},q^{2r-2(d-1)n};q^{2d})_{(dn-n-r)/d}} \nonumber \\&\quad \times \sum _{j_1,\dots ,j_{d+s-1}\geqslant 0} \frac{(q^{-2d};q^{2d})_{j_1}\cdots (q^{-2d};q^{2d})_{j_s}(q^{2(d-r)};q^{2d})_{j_{s+1}}\cdots (q^{2(d-r)};q^{2d})_{j_{d+s-1}}}{(q^{2d};q^{2d})_{j_1}\cdots (q^{2d};q^{2d})_{j_{d+s-1}}} \nonumber \\&\quad \times \frac{(q^{2d+r},q^{2d+r};q^{2d})_{j_1}\cdots (q^{2d+r},q^{2d+r};q^{2d})_{j_1+\cdots +j_{s-1}}}{(q^r,q^r;q^{2d})_{j_1}\cdots (q^r,q^r;q^{2d})_{j_1+\cdots +j_{s-1}}}\nonumber \\&\quad \times \frac{(q^{2r},q^{2r};q^d)_{j_1+\cdots +j_{s}} \dots (q^{2r},q^{2r};q^{2d})_{j_1+\dots +j_{d+s-2}} (q^{2r},q^{2d+2(d-1)n};q^{2d})_{j_1+\dots +j_{d+s-1}}}{(q^{2d},q^{2d};q^{2d})_{j_1+\cdots +j_s} \dots (q^{2d},q^{2d};q^{2d})_{j_1+\dots +j_{d+s-1}}} \nonumber \\&\quad \times \frac{(q^{2r-2(d-1)n};q^{2d})_{j_1+\dots +j_{d+s-1}}}{(q^{2(d+r)};q^{2d})_{j_1+\dots +j_{d+s-1}}}\nonumber \\&\quad \times \frac{q^{2(d+r)(j_{d+s-2}+\dots +(d+s-2)j_1)} q^{2d(j_1+\dots +j_{d+s-1})}}{q^{2(2d+r)j_1}\cdots q^{2(2d+r)(j_1+\cdots +j_s)} q^{4r(j_1+\cdots +j_{s+1})}\cdots q^{4r(j_1+\dots +j_{d+s-2})}}. \end{aligned}$$

It is not difficult to see that the *q*-shifted factorial $(q^{2(d+r)};q^{2d})_{(dn-n-r)/d}$ (appearing in the prefactor of the multi-sum) has the factor $1-q^{2(d-1)n}$ which is divisible by $\Phi _n(q^2)$. Similarly, the *q*-shifted factorial $(q^{-2(d-1)n};q^{2d})_{(dn-n-r)/d}$ has the factor $1-q^{-2(d-1)n}$ (being congruent to 0 modulo $\Phi _n(q^2)$) since $(dn-n-r)/d\geqslant 1$ holds in view of the conditions $d\geqslant 2$, $r\leqslant d-2$, and $n\geqslant d-r$. This shows that the *q*-factorial $(q^{2(d+r)},q^{-2(d-1)n};q^{2d})_{(dn-n-r)/d}$ appearing in the numerator of the prefactor of the multi-sum is congruent to 0 modulo $\Phi _n(q^2)^2$. Moreover, it is clear that the *q*-factorial $(q^{2d},q^{2r-2(d-1)n};q^{2d})_{(dn-n-r)/d}$ in the denominator is coprime with $\Phi _n(q^2)$.

Notice that the non-zero terms in the multi-sum in (2.7) are those with multi-index $(j_1,\ldots ,j_{d+s-1})$ satisfying the conditions $j_1+\dots +j_{d+s-1}\leqslant (dn-n-r)/d$ and $j_1,\ldots ,j_s\leqslant 1$ because the product $(q^{-2d};q^{2d})_{j_1}\cdots (q^{-2d};q^{2d})_{j_s}(q^{2r-2(d-1)n};q^{2d})_{j_1+\dots +j_{d+s-1}}$ appears as a factor in the numerator. None of the factors appearing in the denominator of the multi-sum of (2.7) incorporate a factor of the form $1-q^{2\alpha n}$ (and are consequently coprime with $\Phi _n(q^2)$), except for $(q^{2(d+r)};q^{2d})_{j_1+\dots +j_{d+s-1}}$ when $j_1+\dots +j_{d+s-1}=(dn-n-r)/d$. Let $n=ad-r$ (with $a\geqslant 1$). Consider the case $j_1+\dots +j_{d+s-1}=(dn-n-r)/d=a(d-1)-r$ and $j_1,\ldots ,j_s\leqslant 1$. Then $j_{s+1}+\dots +j_{d+s-1}\geqslant a(d-1)-r-s$. Since $r\leqslant d-s-2$, there must exist an *i* ($s+1\leqslant i\leqslant d+s-1$) such that $j_i\geqslant a$. Then $(q^{2(d-r)};q^{2d})_{j_i}$ contains the factor $1-q^{2(d-r)+2d(a-1)}=1-q^{2n}$ which is a multiple of $\Phi _n(q^2)$. Hence, the reduced denominator of the multi-sum in (2.7) is coprime with $\Phi _n(q^2)$. It remains to prove that the multi-sum in (2.7), ignoring the prefactor, is congruent to 0 modulo $\Phi _n(q^2)^2$.

By repeatedly applying Lemma 2.1, the multi-sum in (2.7), without the prefactor, modulo $\Phi _n(q^2)^2$, is congruent to

$$\begin{aligned}&\sum _{j_1,\dots ,j_{d+s-1}\geqslant 0} \frac{(q^{-2d};q^{2d})_{j_1}\cdots (q^{-2d};q^{2d})_{j_s}(q^{2(d-r)};q^{2d})_{j_{s+1}}\cdots (q^{2(d-r)};q^{2d})_{j_{d+s-1}}}{(q^{2d};q^{2d})_{j_1}\cdots (q^{2d};q^{2d})_{j_{d+s-1}}} \nonumber \\&\quad \times \frac{(q^{2d+r},q^{2d+r};q^{2d})_{j_1}\cdots (q^{2d+r},q^{2d+r};q^{2d})_{j_1+\cdots +j_{s-1}}}{(q^{r},q^{r};q^{2d})_{j_1}\cdots (q^{r},q^{r};q^{2d})_{j_1+\cdots +j_{s-1}}}\nonumber \\&\quad \times \frac{(q^{2r-2(d-1)n},q^{2r+2(d-1)n};q^{2d})_{j_1+\cdots +j_{s}}\dots (q^{2(r-n)},q^{2(r+n)};q^{2d})_{j_1+\dots +j_{d+s-2}} }{(q^{2d+2dn},q^{2d-2dn};q^{2d})_{j_1+\cdots +j_s} \dots (q^{2d+4n},q^{2d-4n};q^{2d})_{j_1+\dots +j_{d+s-2}} } \nonumber \\&\quad \times \frac{(q^{2r},q^{2d+2(d-1)n};q^{2d})_{j_1+\dots +j_{d+s-1}}}{(q^{2d+2n},q^{2d-2n};q^{2d})_{j_1+\dots +j_{d+s-1}}} \frac{(q^{2r-2(d-1)n};q^{2d})_{j_1+\dots +j_{d+s-1}}}{(q^{2(d+r)};q^{2d})_{j_1+\dots +j_{d+s-1}}} \nonumber \\&\quad \times \frac{q^{2(d+r)(j_{d+s-2}+\dots +(d+s-2)j_1)} q^{2d(j_1+\dots +j_{d+s-1})}}{q^{2(2d+r)j_1}\cdots q^{2(2d+r)(j_1+\cdots +j_s)} q^{4r(j_1+\cdots +j_{s+1})}\cdots q^{4r(j_1+\dots +j_{d+s-2})}}. \end{aligned}$$

However, this sum vanishes in light of the $m=d+s$, $q\mapsto q^{2d}$, $a\mapsto q^{2r}$, $e_{s+1}=q^{2d+2(d-1)n}$, $e_{s+i}\mapsto q^{2r+2(d-i+1)n}$ ($2\leqslant i\leqslant d$), $n_1=\cdots =n_{s}=1$, $n_{s+1}=0$, $n_{s+j}\mapsto (n+r-d)/d$ ($2\leqslant j\leqslant d$), $N=(dn-n-r)/d$, case of Lemma 2.2.

This proves that the *q*-hypergeometric congruence (1.5) is true modulo $\Phi _n(q^2)^4$ for $M=(dn-n-r)/d$. Since $n\geqslant d-r$, we have $(dn-n-r)/d\leqslant n-1$. It is clear that $(q^{2r};q^{2d})_k/(q^{2d};q^{2d})_k \equiv 0\pmod {\Phi _n(q^2)}$ for $(dn-n-r)/d<k\leqslant n-1$. Thus, we see that (1.5) is also true modulo $\Phi _n(q^2)^4$ for $M=n-1$. $\square$

## Proof of (1.5) modulo $[n]_{q^2}$

We need the following easily proved result, which first appeared in [14, Lemma 2.1].

### Lemma 3.1

Let *d*, *m* and *n* be positive integers with $m\leqslant n-1$. Let *r* be an integer satisfying $dm\equiv -r\pmod {n}$. Then, for $0\leqslant k\leqslant m$,

$$\begin{aligned} \frac{(aq^r;q^d)_{m-k}}{(q^d/a;q^d)_{m-k}} \equiv (-a)^{m-2k}\frac{(aq^r;q^d)_k}{(q^d/a;q^d)_k} q^{m(dm-d+2r)/2+(d-r)k} \pmod {\Phi _n(q)}. \end{aligned}$$

In order to prove that (1.5) is true modulo $[n]_{q^2}$, we shall establish the following more general result. It is easy to see that the condition $n\equiv -r\pmod {d}$ guarantees that $m=(dn-n-r)/d$ is an integer.

### Theorem 3.2

Let *d*, *n* be positive integers with $\gcd (d,n)=1$. Let *r*, *s* be integers with $s\geqslant 0$. Then

3.1 $$\begin{aligned} \sum _{k=0}^{m}[2dk+r]_{q^2} [2dk+r]^{2s} \frac{(q^{2r};q^{2d})_k^{2d}}{(q^{2d};q^{2d})_k^{2d}}q^{2d(d-r-s-1)k} \equiv 0\pmod {[n]_{q^2}}, \end{aligned}$$

and

3.2 $$\begin{aligned} \sum _{k=0}^{n-1}[2dk+r]_{q^2} [2dk+r]^{2s} \frac{(q^{2r};q^{2d})_k^{2d}}{(q^{2d};q^{2d})_k^{2d}}q^{2d(d-r-s-1)k} \equiv 0\pmod {[n]_{q^2}}, \end{aligned}$$

where $$0\leqslant m\leqslant n-1$$ and $$dm\equiv -r\pmod {n}$$.

### Proof

Since $\gcd (d,n)=1$, there exists a non-negative integer $m\leqslant n-1$ such that $dm\equiv -r\pmod {n}$. By the $a=1$ and $q\mapsto q^2$ case of Lemma 3.1, we can easily verify that, for $0\leqslant k\leqslant m$, the *k*-th and $(m-k)$-th terms on the left-hand side of (3.1) cancel each other modulo $\Phi _n(q)$, i.e.,

$$\begin{aligned}&[2d(m-k)+r]_{q^2} [2d(m-k)+r]^{2s} \frac{(q^{2r};q^{2d})_{m-k}^{2d}}{(q^{2d};q^{2d})_{m-k}^{2d}}q^{2d(d-r-s-1)(m-k)} \\&\quad \equiv -[2dk+r]_{q^2} [2dk+r]^{2s} \frac{(q^{2r};q^{2d})_k^{2d}}{(q^{2d};q^{2d})_k^{2d}}q^{2d(d-r-s-1)k} \pmod {\Phi _n(q^2)}. \end{aligned}$$

This immediately leads to the *q*-congruence (3.1) modulo $\Phi _n(q^2)$. Furthermore, since $dm\equiv -r\pmod {n}$, for $m< k\leqslant n-1$ the *q*-shifted factorial $(q^{2r};q^{2d})_k$ contains the factor $1-q^{2r+2dm}=1-q^{2an}$ (*a* is an integer) and is therefore congruent to 0 modulo $\Phi _n(q^2)$. At the same time, the *q*-shifted factorial $(q^{2d};q^{2d})_k$ is coprime with $\Phi _n(q^2)$ for $m< k\leqslant n-1$. Thus, each summand in (3.2) with *k* in the range $m< k\leqslant n-1$ is congruent to 0 modulo $\Phi _n(q^2)$. This, along with (3.1), establishes the *q*-congruence (3.2) modulo $\Phi _n(q^2)$.

Let $\zeta \ne 1$ be an *n*-th root of unity. That is, $\zeta$ is a primitive root of unity of odd degree $n_1$ such that $n_1\mid n$. Let $c_q(k)$ stand for the *k*-th term on the left-hand side of (3.2). Specifically,

$$\begin{aligned} c_q(k)=[2dk+r]_{q^2} [2dk+r]^{2s} \frac{(q^{2r};q^{2d})_k^{2d}}{(q^{2d};q^{2d})_k^{2d}}q^{2d(d-r-s-1)k}. \end{aligned}$$

Now the *q*-congruences (3.1) and (3.2) modulo $\Phi _n(q^2)$ with $n=n_1$ imply that

$$\begin{aligned} \sum _{k=0}^{m_1}c_{\zeta }(k)&=\sum _{k=0}^{n_1-1}c_{\zeta }(k)=0, \end{aligned}$$

and

$$\begin{aligned} \sum _{k=0}^{m_1}c_{-\zeta }(k)&=\sum _{k=0}^{n_1-1}c_{-\zeta }(k)=0, \end{aligned}$$

where $0\leqslant m_1\leqslant n_1-1$ and $dm_1\equiv -r\pmod {n_1}$. Noticing that, for all non-negative integers $\ell$ and *k*,

$$\begin{aligned} \frac{c_\zeta (\ell n_1+k)}{c_\zeta (\ell n_1)} =\lim _{q\rightarrow \zeta }\frac{c_q(\ell n_1+k)}{c_q(\ell n_1)} =\frac{c_\zeta (k)}{[r]_{\zeta ^2}[r]_\zeta ^{2s}}, \end{aligned}$$

we have

3.3 $$\begin{aligned} \sum _{k=0}^{n-1}c_\zeta (k)=\sum _{\ell =0}^{n/n_1-1} \sum _{k=0}^{n_1-1}c_\zeta (\ell n_1+k) =\frac{1}{[r]_{\zeta ^2}[r]_\zeta ^{2s}}\sum _{\ell =0}^{n/n_1-1}c_\zeta (\ell n_1) \sum _{k=0}^{n_1-1}c_\zeta (k)=0, \end{aligned}$$

and

$$\begin{aligned} \sum _{k=0}^{m}c_\zeta (k) =\frac{1}{[r]_{\zeta ^2}[r]_\zeta ^{2s}}\sum _{\ell =0}^{(m-m_1)/n_1-1} c_\zeta (\ell n_1)\sum _{k=0}^{n_1-1}c_\zeta (k) +\frac{c_\zeta (m-m_1)}{[r]_{\zeta ^2}[r]_\zeta ^{2s}}\sum _{k=0}^{m_1}c_\zeta (k)=0. \end{aligned}$$

This proves that the two sums $\sum _{k=0}^{m}c_q(k)$ and $\sum _{k=0}^{n-1}c_q(k)$ are congruent to 0 modulo $\Phi _{n_1}(q)$. Along the same lines we may prove that they are also congruent to 0 modulo $\Phi _{n_1}(-q)$. Letting $n_1$ run over all divisors of *n* greater than 1, we deduce that these two sums are congruent to 0 modulo

$$\begin{aligned} \prod _{n_1\mid n,\, n_1>1}\Phi _{n_1}(q) \Phi _{n_1}(-q)=[n]_{q^2}, \end{aligned}$$

thus establishing (3.1) and (3.2). $\square$

## Two open problems

It seems that, for $r=-1$ and $p\equiv 1\pmod {d}$, the following stronger version of (1.6) is true.

### Conjecture 4.1

Let *d*, *s* be integers satisfying $d\geqslant 2$ and $0\leqslant s\leqslant d-1$. Let *p* be a prime with $p\equiv 1\pmod {d}$. Then, for $m\geqslant 1$,

4.1 $$\begin{aligned} \sum _{k=0}^{M}(2dk-1)^{2s+1} \frac{(-\frac{1}{d})_k^{2d}}{(1)_k^{2d}} \equiv 0 \pmod {p^{4m}}, \end{aligned}$$

where $$M=(dp^m-p^m+1)/d$$ or $$M=p^m-1$$.

Furthermore, we believe the following *q*-analogue of (4.1) should also be true.

### Conjecture 4.2

Let *d*, *s* be integers satisfying $d\geqslant 2$ and $0\leqslant s\leqslant d-1$. Let $n\geqslant d+1$ be an integer with $n\equiv 1\pmod {d}$. Then, for $m\geqslant 1$,

4.2 $$\begin{aligned} \sum _{k=0}^{M}[2dk-1]_{q^2} [2dk-1]^{2s} \frac{(q^{-2};q^{2d})_k^{2d}}{(q^{2d};q^{2d})_k^{2d}}q^{2d(d-s)k} \equiv 0 \left( \textrm{mod}\,{\prod _{i=1}^m \Phi _{n^i}(q)^4}\right) , \end{aligned}$$

where $$M=(dn^m-n^m+1)/d$$ or $$M=n^m-1$$.

Since $\Phi _{p^i}(1)=p$ for any prime *p* and positive integer *i*, the supercongruence (4.1) follows from (4.2) by taking $n=p$ and $q\rightarrow 1$. Applying Zeilberger’s algorithm [20] and its *q*-analogue, the authors [10] have proved that, for any odd integer $n>1$,

$$\begin{aligned} \sum _{k=0}^{M}[4k-1]\frac{(q^{-1};q^2)_k^4}{(q^2;q^2)_k^4} q^{4k} \equiv -(1+3q+q^2)[n]^4 \pmod {[n]^4\Phi _n(q)}, \end{aligned}$$

where $M=(n+1)/2$ or $M=n-1$. Replacing *n* by $n^m$ and *q* by $q^2$ in the above *q*-supercongruence, and noticing that $[n^m]_{q^2}$ contains the factor $\prod _{i=1}^m \Phi _{n^i}(q)$, we see that Conjecture 4.2 is true for $d=2$ and $s=0$.

## Data Availability

Data sharing not applicable to this article as no datasets were generated or analysed during the current study.
